# Effect of SWI/SNF chromatin remodeling complex on HIV-1 Tat activated transcription

**DOI:** 10.1186/1742-4690-3-48

**Published:** 2006-08-07

**Authors:** Emmanuel Agbottah, Longwen Deng, Luke O Dannenberg, Anne Pumfery, Fatah Kashanchi

**Affiliations:** 1 The George Washington University Medical Center, Department of Biochemistry and Molecular Biology, Washington, DC 20037, USA; 2 Seton Hall University, Department of Biology, South Orange, NJ 07079, USA; 3 The Institute for Genomic Research (TIGR), Rockville, MD 20850, USA

## Abstract

**Background:**

Human immunodeficiency virus type 1 (HIV-1) is the etiologic agent of acquired immunodeficiency virus (AIDS). Following entry into the host cell, the viral RNA is reverse transcribed into DNA and subsequently integrated into the host genome as a chromatin template. The integrated proviral DNA, along with the specific chromatinized environment in which integration takes place allows for the coordinated regulation of viral transcription and replication. While the specific roles of and interplay between viral and host proteins have not been fully elucidated, numerous reports indicate that HIV-1 retains the ability for self-regulation via the pleiotropic effects of its viral proteins. Though viral transcription is fully dependent upon host cellular factors and the state of host activation, recent findings indicate a complex interplay between viral proteins and host transcription regulatory machineries including histone deacetylases (HDACs), histone acetyltransferases (HATs), cyclin dependent kinases (CDKs), and histone methyltransferases (HMTs).

**Results:**

Here, we describe the effect of Tat activated transcription at the G_1_/S border of the cell cycle and analyze the interaction of modified Tat with the chromatin remodeling complex, SWI/SNF. HIV-1 LTR DNA reconstituted into nucleosomes can be activated *in vitro *using various Tat expressing extracts. Optimally activated transcription was observed at the G_1_/S border of the cell cycle both *in vitro *and *in vivo*, where chromatin remodeling complex, SWI/SNF, was present on the immobilized LTR DNA. Using a number of *in vitro *binding as well as *in vivo *chromatin immunoprecipitation (ChIP) assays, we detected the presence of both BRG1 and acetylated Tat in the same complex. Finally, we demonstrate that activated transcription resulted in partial or complete removal of the nucleosome from the start site of the LTR as evidenced by a restriction enzyme accessibility assay.

**Conclusion:**

We propose a model where unmodified Tat is involved in binding to the CBP/p300 and cdk9/cyclin T_1 _complexes facilitating transcription initiation. Acetylated Tat dissociates from the TAR RNA structure and recruits bromodomain-binding chromatin modifying complexes such as p/CAF and SWI/SNF to possibly facilitate transcription elongation.

## Background

Human immunodeficiency virus (HIV) is the etiological agent of AIDS. The pathogenesis of HIV-induced disease is complex and multifactorial [1]. Following infection, reverse transcriptase complexes synthesize a double stranded DNA molecule that is then incorporated into the host genome. A robust cellular and humoral immune response inhibits viral production within weeks. However, a chronic persistent infection in lymphoid tissue persists throughout the life (median period of 10–20 years) of the infected individual. Several key HIV-1 and cellular proteins have been determined to be necessary for this course of infection, including the *trans*-activator Tat. Viral clones deficient in Tat do not effectively replicate *in vitro *or *in vivo*. Furthermore, infected T cells quiescent at the G_0 _phase of the cell cycle (lacking cytokine signals) will not produce high titer virus [2].

The replication rate of integrated HIV-1 is largely controlled at the level of transcription. The HIV-1 LTR, present at both ends of the integrated viral genome, contains *cis*-acting elements necessary for transcription initiation from the 5' LTR and for polyadenylation of the viral transcripts in the 3' LTR. The core promoter of HIV-1 includes two NF-κB binding sites, three Sp1 binding sites, the TATA box, and the ligand-binding protein 1 (LBP-1)/YY1 site. There is also a repressor complex sequence (RCS) within the initiation site, which contains three binding motifs for LSF. YY1 and LSF cooperate to allow binding of YY1 to the RCS and subsequent recruitment of HDAC1 [3]. In addition to cellular transcription factors, the activity of the HIV-1 promoter is strongly dependent on the viral *trans*-activator, Tat. Historically, the mechanism of action by Tat has been assigned to be at the level of initiation and elongation [4-9]. The effect of Tat on pre-initiation, initiation, and elongation has been observed through a number of biochemical interactions, including physical binding to Sp1[10], stabilization of the TFIID/TFIIA complex on the HIV-1 TATA box [11], recruitment of a functional TATA-binding protein (TBP) or TFIID [11-16], phosphorylation of the carboxy-terminal domain (CTD) of RNA polymerase II (RNAPII) by a number of kinases, including TFIIH [17-19], hSpt5 which functions in transcription elongation as a stabilization factor of RNAPII [20], and binding of Tat directly to RNAPII [21,22]. More recently the role of Tat in transcription initiation has been revisited, where Tat enhanced recruitment of TBP has been observed resulting in activated transcription [23,24].

Tat activates the HIV LTR by binding to TAR and recruiting and activating cellular factors [25-31]. One of the co-factors required for Tat activity is a protein kinase called TAK (Tat-associated kinase) [32,33] whose activity is stimulated by Tat. Activation of TAK (cdk9/cyclin T_1_) results in hyper-phosphorylation of the large subunit of the RNAPII CTD and activation of transcription elongation [34]. Cdk9 is analogous to a component of a positive acting elongation factor (P-TEFb) isolated from Drosophila [35], which acts to stimulate promoter-paused RNAPII to enter into productive elongation [33,36-39]. A histidine-rich stretch of cyclin T_1 _binds to the CTD of RNAPII, which is required for the subsequent expression of full-length transcripts from target genes [40]. Cdk9 phosphorylation is required for high-affinity binding of Tat/P-TEFb to TAR [41-43].

HIV-1 proviral DNA is organized into a higher order chromatin structure *in vivo*, which regulates viral expression by restricting access of the transcriptional machinery to the HIV-1 LTR. The primary chromatin structure of integrated HIV-1 has been characterized *in vivo *by the DNase-1 digestion method [44-46]. Two chronically infected human cell lines, ACH_2 _(T-cell line) and U_1 _(promonocyte cell line), were analyzed by DNase-I digestion and four distinct DNase-I hypersensitive sites (DHS) were identified in the 5' LTR. It has been largely assumed that DHSs in the native chromatin are free of histones and allow unrestricted access to DNA-binding proteins [47]. The first site, DHS1, located at the 5' end of the integrated LTR, was detected only as a minor DHS in U_1 _cells. DHS2 and DHS3 are located in the U3 region, encompassing nt 223–325 and nt 390–449, respectively. The NF-κB and Sp1 binding sites, as well as the TATA box, are located in DHS2 and DHS3. DHS4 was found immediately downstream of the U5 region (nt 656–720), where a cluster of potential transcription factor binding sites for AP-1, AP-3 like, DBF-1, and Sp1 are positioned. The location of the DHSs was also verified *in vitro *using the HIV-1 promoter reconstituted into chromatin by DNase-I footprinting analysis [48-50]. Furthermore, independent of the viral integration site, five nucleosomes (nuc-0 to nuc-4) are precisely positioned within the 5' LTR. In the transcriptionally silent provirus, these nucleosomes define two large nucleosome-free regions spanning nt -255 to -3 and +141 to +265. One nucleosome, nuc-1, is located between these two regions. The first nucleosome-free region in U3 contains many promoter/enhancer elements which are already occupied by transcriptional factors including repressors [51]. When cells are activated with TNF-α and TPA treatment or with the HDAC-specific inhibitors, trapoxin, Trichostatin A (TSA), or sodium butyrate, DHS3 and DHS4 are extended and nuc-1 is specifically remodeled. Using a ChIP assay, histone acetylation surrounding nuc-1 and the RCS was observed to be significantly increased following TSA treatment [52]. The disruption of nuc-1 occurred independent of transcription and was α-amanitin insensitive, suggesting that this chromatin remodeling was a pre-requisite for transcription, rather than a consequence [46]. Therefore, the integrated proviral DNA, along with the specific chromatinized environment in which integration takes place allows for the coordinated regulation of viral transcription and replication. While the specific roles of and interplay between viral and host proteins have not been fully elucidated, numerous reports indicate that HIV-1 retains the ability for self-regulation via the pleiotropic effects of its viral proteins. Though viral transcription is fully dependent upon host cellular factors, recent findings indicate a complex interplay between viral proteins and host transcription and signaling machineries (i.e. HDACs, HATs, and other protein-modifying factors).

In the current manuscript, we describe the effect of Tat activated transcription at the G_1_/S border of the cell cycle and analyze the interaction of Tat with the chromatin remodeling complex, SWI/SNF. HIV-1 LTR DNA reconstituted into nucleosomes can be activated *in vitro *using various Tat expressing extracts. More specifically, optimal activated transcription was observed at the G_1_/S border of the cell cycle both *in vitro *and *in vivo*. In an attempt to define protein complexes that are normally involved in HIV-1 transcription, we used an *in vitro *immobilized nucleosomal DNA assembly system in the presence of the G_1_/S extracts and pulled-down complexes followed by their identification using MALDI-TOF mass spectrometry. In addition to known factors such as Sp1, TFIIB, and cdk9/cyclin T_1_, we also specifically detected the presence of acetylated Tat and members of the SWI/SNF chromatin remodeling complex. Using a number of *in vitro *binding as well as *in vivo *ChIP assays, we detected the presence of both BRG1 and acetylated Tat in the same complex. Finally, we demonstrate that removal of nuc-1 resulted in accessibility of the HIV-1 LTR DNA to a restriction enzyme and activated transcription.

## Results

### Effect of Tat activation at the G_1_/S border

We previously demonstrated, by *in vitro *transcription analysis, that Tat-dependent transcription takes place in a cell cycle-dependent manner. We utilized cells that were transfected with either a control plasmid (pCEP4) or a hemagglutinin epitope-tagged Tat plasmid (eTat), selected in hygromycin by single-cell dilution, and maintained in hygromycin. Whole-cell extracts were prepared from control and eTat cells for *in vitro *transcription assays using wild-type and mutant HIV-1 naked templates. Cells at late G_1_/early S contained 10-fold higher levels of transcriptional activity on the wild-type LTR template (LTR-TAR^+^) than on the TAR mutant template (LTR-TAR^-^) [53].

**Figure 1:**
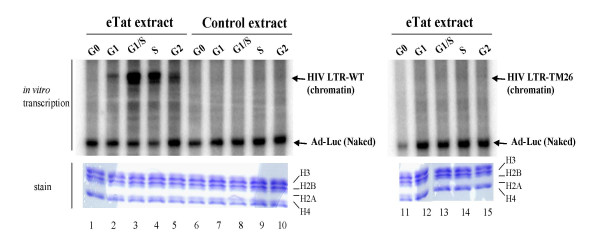
**HIV chromatin transcription at various stages of the cell cycle**. To obtain synchronized extracts, both eTat and control cells were first treated with hydroxyurea, released and then treated with nocodazole. Extracts were made from 1 h (G_0_), 3 h (early G_1_), 6 h (G_1_/S), 15 h (late S), and 21 h (G_2_) post-nocodazole release and used in an *in vitro *transcription reaction. Released cells (1/10) were processed for FACS profile (data not shown). Two micrograms of chromatin DNA were used in each reaction. The bottom panels are gels of histones from immobilized DNA after transcription stained with Coomasie Blue. Lanes 1–5 and 11–15 are using eTat extract, and lanes 6–10 are using control pCEP4 extract. The left panel contains HIV-LTR-TAR wild-type and HIV-LTR-TAR mutant (TM26), and 200 ng of naked DNA (AdLuc, [55]).

Our previous results were performed with HIV-1 naked LTR DNA; however, *in vivo*, the HIV-1 DNA is assembled into organized nucleosomal DNA and the presence of nucleosomes generally acts as an inhibitor of transcription. Therefore, to closely mimic the *in vivo *scenario, we decided to reconstitute HIV-1 LTR DNA into nucleosomes and use the templates in an *in vitro *transcription reaction. Nucleosomes were assembled on the HIV-1 LTR as previously described [48,54,55]. Following reconstitution, extracts from various stages of cell cycle were used in an *in vitro *transcription reaction. To obtain a uniform population of cells, we treated both eTat and control cells first with hydroxyurea, released, treated with nocodazole, followed by release at various time points. Extracts were made from the control and eTat cell lines at G_0_, early G_1_, G_1_/S, late S, and G_2 _for *in vitro *transcription. Three types of DNA were used in these assays, including HIV-LTR-TAR wild-type, HIV-LTR-TAR mutant (TM26) (both assembled into chromatin) and Adenovirus-Luc plasmid (AdLuc) as a naked DNA control [55]. Transcription from the WT HIV LTR was first observed with eTat extracts purified from the G_1 _phase (Figure 1, left panel, lane 2). Transcription was maximal at the G_1_/S border and declined during the S and G_2 _phases (lanes 4 and 5). Very little detectable transcription was observed with extracts obtained at the G_0 _(lane 1) or in control extracts. Furthermore, no transcription was observed when a TAR mutant template (TM26) was used (lanes 11–15). These results indicate that HIV-1 transcription started in the early G_1 _phase (lane 2); however, a more robust activity was observed at the G_1_/S border (lane 3). In all cases ample transcription was observed using naked AdLuc DNA. All HIV-1 DNAs still contained core histones after transcription, indicating that nucleosomes were still present (bottom panel). These results indicate that robust Tat activated transcription begins at the G_1_/S border of the cell cycle.

### Presence of HIV-1 transcripts at the G1/S border in infected cells

**Figure 2:**
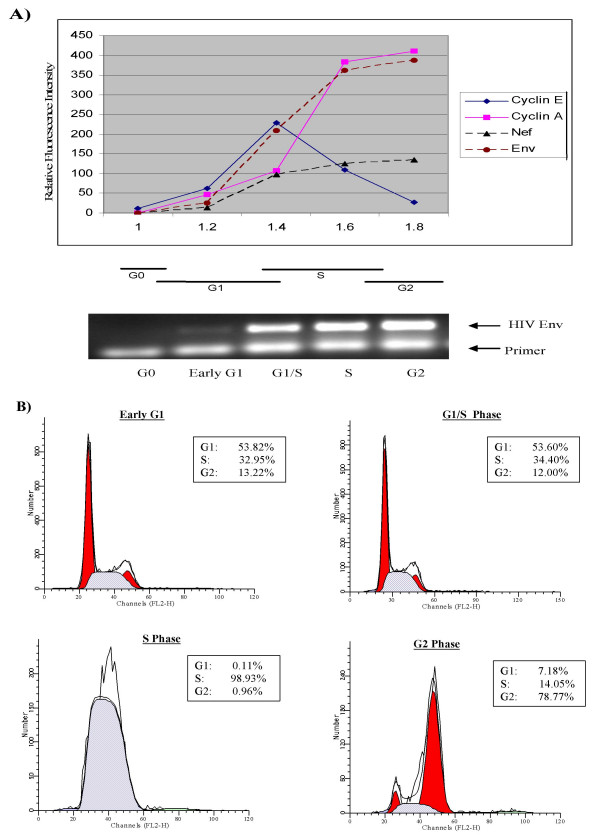
**HIV-1 Nef and Env expression in individual cells at various phases of the cell cycle**. **A) **HIV-1 protein expression was monitored by flow cytometric analysis. OM10.1 cells were washed in Hanks' balanced salt solution, fixed in 70% ice-cold ethanol, and stained with mouse anti-human monoclonal antibodies to cyclin E, cyclin A, Nef, Env, or a non-specific immunoglobulin (DAKO) overnight. Cells in G_1 _and G_2 _were identified by DNA content and divided the intervening region into 10 to 12 equal increments to allow the calculation of mean protein-associated fluorescence and mean DNA content. The exact same analysis was performed on the appropriate aliquot stained with non-specific immunoglobulin. Protein fluorescence was determined by subtracting the mean from the nonspecific samples. Samples were washed in PBS-BSA and stained with a fluorescein isothiocyanate-labeled goat anti-mouse secondary antibody (1:30; DAKO) for 30 min in the dark. The bottom of panel A shows HIV-1 gene expression in G_0_, early G_1_, G_1_/S, S, and G_2 _phases of cell cycle by RT-PCR with primers for Env and Nef [58, 59]. **B) **Cell cycle analysis of cells after G_0 _release. Cells were synchronized at G_0 _by serum starvation and samples were removed from the medium at each time point, washed with PBS without Mg^2+ ^or Ca^2+^, fixed with 70% ethanol, and stained with propidium iodide followed by cell-sorting analysis on a Coulter EPICS cell analyzer. The FACS profile for each time point is presented along with the percentage of cells in G_1_, S, and G_2 _(upper right side boxes of each histogram).

To determine the timing of HIV-1 transcription, we measured the accumulation of two HIV-1 proteins (Nef and Env), as well as cyclin E and cyclin A (positive controls) in OM10.1 cells during various stages of the cell cycle. We measured these proteins by a multiparametric flow cytometric approach [56]. This technique allowed us to examine the accumulation of multiple proteins in individual cells, as opposed to immunoblotting, which determines the accumulation of proteins in a large, diverse cell population. Furthermore, the flow cytometric approach allows us to correlate this value with respect to the cell's position in the cycle by measuring DNA content and multiple analyses can be carried out in the same cell with different fluorescent markers. OM10.1 cells are a promyelocytic line containing a transcriptionally latent, single copy of wild-type HIV-1 integrated proviral DNA [57]. For cell cycle analysis, these cells were initially arrested at G_0 _by feeding with serum starvation medium for three days. Cells were subsequently treated with TNF-α (10 ng/ml) for 2 h to induce virus, washed, and incubated at 37°C with complete medium. Samples (2/3) were collected at various time points and analyzed using flow cytometry. The remaining samples (1/3) were processed for RT-PCR using primers for Env and Nef [58,59]. The positive controls, cyclin E (hallmark of G_1_/S transcription) and cyclin A (hallmark of S transcription), were detectable in OM10.1 cells (Figure 2A). More importantly, HIV-1 proteins, namely Nef (from a doubly spliced transcript) and Env (from a singly spliced transcript), were detected starting at the G_1_/S phase of the cell cycle. RT-PCR assays show that these samples display robust Env transcription at the G_1_/S border (Figure 2A, bottom panel). Propidium iodide staining of host DNA from various cell populations used in antibody staining and RT-PCR analysis (Figure 2B) demonstrated that the samples used in Figure 2A were at particular phases of the cell cycle. These results are also consistent with our previous work where we showed that HLM-1 cells (HIV-1^+^/Tat^-^, [60]), which contain a single copy of full-length HIV-1 provirus with a triple termination codon at the first AUG of the Tat gene, had dramatically reduced levels of Gag/p24 antigen production when arrested at G_1_/S [61].

### Presence of chromatin remodeling factors on HIV-1 DNA at the G_1_/S border

**Figure 3:**
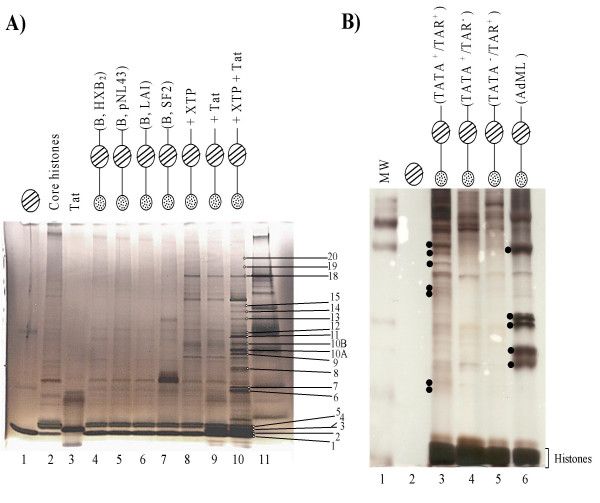
**Identification of cellular proteins associated with the transcriptionally active nucleosomal HIV-1 promoter**. **A) **Nucleosomes were assembled on biotin labeled HIV-1 LTR DNA and G_1_/S extracts were added to the reaction mixture. Following washes (TNE_600 _plus 1% NP-40), samples were eluted with excess biotin and centrifuged to pellet the beads. Supernatants were TCA precipitated and ran on a 4–20% gel and silver stained (MALDI compatible). Lane 1, Strepavidin beads; lane 2, purified core histones before nucleosomal assembly; lane 3, purified Tat; lanes 4–7 are LTR sequences (-110/+180) from four different clade B isolates assembled and incubated with G_1_/S extracts without any nucleotide addition; lane 8, transcriptionally active HXB_2 _promoter with nucleotide addition (ATP, CTP, GTP); lane 9, HXB_2 _promoter with purified Tat; lane 10 same as lane 8 with purified Tat; lane 11, the rainbow molecular marker (14–220 kDa). **B) **Control gel utilizing HXB_2 _wild-type, TATA^-^/TAR^+^, and TATA^+^/TAR^- ^promoters with Tat and nucleotide (ATP, CTP, GTP) and AdML promoters. Unique proteins (**.**) bound to DNA were cut out, trypsin digested, and used for identification by mass spectrometry [74].

To define some of the protein complexes that are normally involved in HIV-1 transcription, we utilized an *in vitro *nucleosomal DNA assembly system, where the DNA was labeled with biotin and bound to strepavidin beads [62]. Subsequently, G_1_/S extracts were mixed with the nucleosomal DNA and unbound material was washed away. Retained complexes were separated on a 4–20% SDS/PAGE and unique bands were used for MALDI-TOF mass spectrometry (Figure 3). Four clade B LTR promoters (nt -110 to +180) were used as controls, HXB_2_, pNL43, LAI, and SF2. Also, nucleosomal templates were either transcriptionally inactive (no nucleotides) or were transcriptionally active with three nucleotides added to the reaction (ATP, CTP, and GTP). In the latter case, the polymerase normally stalls at the early stages of elongation. As a further set of controls, we utilized the clade B LTR of wild-type, TATA^+^/TAR^-^, TATA^-^/TAR^+^, and AdML promoters [53].

A limited number of proteins bound to the transcriptionally inactive control promoters including histones H4, H2A, H2B, and H3 (Figure 3A, lanes 4–7). Twenty proteins that bound to the transcriptionally active HIV-1 LTR were identified (Figure 3A, lane 10). The proteins identified included: 1) histone H4; 2) Tat; 3) histone H2A; 4) histone H2B; 5) histone H3; 6) TFIIB; 7) cdk2; 8) cdk9; 9) undetermined; 10A) cyclin E; 10B) cyclin E; 11) cyclin T; 12) undetermined; 13) undetermined; 14) undetermined; 15) undetermined; 16) Sp1; 17) RNA helicase A; 18) RNAPII large subunit; 19) BRG1; and 20) DNA-PK. Additionally, we performed control reactions using HXB_2 _TATA^-^/TAR^+^, HXB_2 _TATA^+^/TAR^-^, and AdML promoters (Figure 3B). A number of unique proteins were bound to the wild-type promoter that were not bound on the HXB_2 _TATA^-^/TAR^+ ^and TATA^+^/TAR^- ^promoters (data not shown). Results in Figure 3A confirms published data showing that TFIIB, cdk2, cdk9, cyclin E, cyclin T_1_, Sp1, RNAPII, and DNA-PK associate with the HIV-1 LTR during basal transcription or Tat activated transcription. These results also showed that cdk9/cyclin T_1 _and other cyclin/cdk complexes (i.e. cdk2/cyclin E) are involved in transcription of the HIV-1 promoter. Finally, these results indicate that Tat recruits a component of the chromatin remodeling complex, BRG1, to the HIV-1 LTR.

### Effect of acetylated Tat on recruitment of a chromatin remodeling complex

The identification of BRG1 as one of the proteins that bound to transcriptionally active HIV-1 LTR suggests that this component of the SWI/SNF complex may be important in Tat activated transcription. To elucidate the role of BRG1 in HIV-1 gene expression, we determined whether there was a direct association with modified Tat. While the detailed molecular mechanisms underlying Tat dissociation from TAR RNA and its *trans*-activation of transcription on the integrated HIV-1 genome remain elusive, increasing evidence suggests that Tat activity requires association with several multiprotein complexes, which include the cyclin T_1_/cdk9 complex [33,36,43,63-69] and the HAT transcriptional coactivators, p300/CBP and p300/CBP-associated factor (p/CAF) [60,70,71]. The site of acetylation of Tat was mapped to a double-lysine motif in a highly conserved region (^49^RKKRRQ^54^) of the basic RNA-binding motif of Tat. Tat acetylation resulted in its dissociation from TAR RNA and promoted the formation of a multiprotein complex comprised of Tat and p/CAF [60,70]. Furthermore, we previously had shown that immobilized biotin Tat or acetylated Tat at positions 41, 50, and 51 could selectively pull-down distinct functional complexes including transcription-associated proteins, acetyltransferase-associated proteins, and kinase proteins [72].

**Figure 4:**
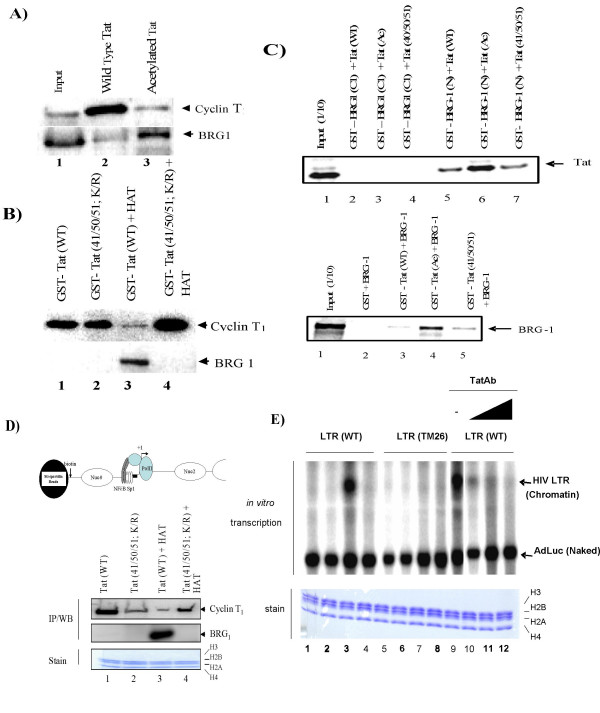
**BRG1 binds to acetylated Tat**. **A) **CEM G_1_/S cell extracts (2 mg) were mixed with 100 μg of biotin-Tat peptides (aa 42–54), incubated for 2 h, and washed. Bound proteins were separated on a 4–20% SDS/PAGE and Western blotted. Lane 1, input; lane 2, unacetylated Tat peptide; and lane 3, acetylated Tat peptide. **B) **GST-Tat (wild-type and mutant) was purified over a glutathione column and eluted. Acetylated GST-Tat was incubated with p300 and acetyl-CoA (62), washed, and incubated with cyclin T_1_/cdk9 (top) or SWI/SNF (bottom). Bound complexes were run on a 4–20% SDS/PAGE and Western blotted for the presence of cyclin T_1 _or BRG1. Lane 1, wild-type Tat; lane 2, Tat with a lysine to arginine change at residues 41, 50, and 51; lane 3, wild-type Tat and p300; lane 4, mutated Tat and p300. **C) **GST proteins and TNT lysates containing ^35^S-labeled Tat or BRG1 were incubated for 2 h at 4°C. Complexes were centrifuged; bound labeled proteins were denatured, subjected to SDS-PAGE, dried, and autoradiographed.** D) **Top: Schematic of the biotinylated HIV-1 LTR DNA used in the pull-down assay. Bottom: Immobilized chromatin HIV-1 LTR templates were incubated with CEM extract and wild-type or mutated Tat, then acetylated in the presence of GST-HAT. Samples were incubated with 100 ng SWI/SNF and all four cold nucleotides. Templates were washed and proteins were separated on a 4–20% SDS/PAGE for Western blot analysis. Bottom: remaining histones after transcription. **E) **CEM cells were treated with hydroxyurea and nocodazole, and samples were processed at 9 h post-release. The extracts were supplemented with purified SWI/SNF (all lanes), plus wild-type Tat (lanes 2 and 6), acetylated Tat (lanes 3 and 7), and Tat mutated at positions 50/51 (lanes 4 and 8). Anti-acetyl Tat 50/51 antibodies were added at time zero (lanes 10–12). Bottom: histone stain from immobilized DNA after transcription.

We confirmed by Western blotting that the unmodified peptide of Tat (aa 42–54) bound cyclin T_1 _while the acetylated form of the Tat peptide bound BRG1. These results were confirmed using full length Tat protein (Figures 4A and 4B). Additionally, we confirmed that acetylated lyines 41, 50, and 51 of Tat are important for BRG1 binding (Figure 4B, lanes 3 and 4). Results presented in panel A used CEM extracts and results in panel B used purified cyclin T_1_/cdk9 and SWI/SNF complexes (containing BRG1). To further validate these findings, we decided to perform an *in vitro *binding assay using ^35^S-Tat or ^35^S-BRG1. Two independent BRG1 domains were incubated with ^35^S-Tat *in vitro*. BRG1-N spans the N-terminal residues 1–282 and shows high sequence divergence from the corresponding region of BRM. BRG1-C1 contains a 99 base pair exon that is unique to BRG1, the conserved E7 sequence, and a portion of the lysine-arginine region [73]. Results in panel C indicate that acetylated Tat was able to bind efficiently to the BRG1-N terminal construct (lane 6) and not the C-terminal domain (lane 3). Unacetylated Tat did not bind BRG1 as efficiently as acetylated Tat (compare lanes 5 and 7 to lane 6). We next performed the reverse experiment, where GST-Tat was allowed to bind to ^35^S-BRG1 *in vitro*. As expected, the acetylated GST-Tat bound to wild-type BRG1, while unacetylated Tat (wild-type or 41/50/51 mutant) bound Tat much less efficiently (lanes 3 and 5, respectively).

**Figure 5:**
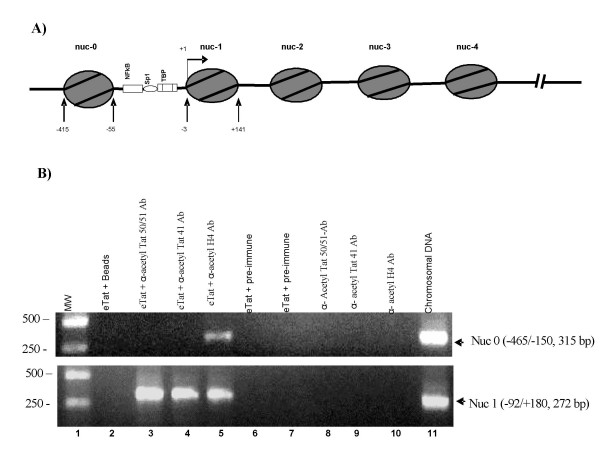
**Acetylated Tat associates with a chromatinized HIV-1 promoter at or near nuc-1 but not nuc-1**. **A) **Diagram of nucleosomes positioned on the integrated HIV-1 genome. The transcription start site is indicated as +1. Critical transcription factor binding sites (NF-κB, Sp1, and TBP) are indicated. Location of nuc-0 through nuc-4 are indicated above the diagram. **B) **ChIP analysis of the HIV-1 genome. The ChIP assay was performed as described [110, 111] with modifications (see Materials and Methods). HLM-1 cells (Tat^-^) [112] were transfected with the pCEP4/eTat vector [53] or no DNA and ChIP analysis was performed with anti- acetyl Tat 50/51 antibodies, anti-acetyl Tat 41 antibodies, or anti-acetyl H4 antibodies (positive control for acetylation of histones and association of nucleosomes with the HIV-1 promoter). The PCR primer pair location and expected sizes are indicated next to the expected amplified PCR products.

To determine whether these complexes bind HIV-1 DNA, we utilized an *in vitro *transcription/assembly system [62], where HIV-1 proviral LTR DNA was assembled into nucleosomal DNA prior to *in vitro *transcription. Nucleosomes were first assembled on biotinylated HIV-1 LTR DNA template and then immobilized using strepavidin agarose beads. The immobilized chromatin LTR was washed in acetylation buffer and used as a substrate for acetylation by p300 in the presence or absence of Tat. Templates were then washed in transcription buffer and used in an *in vitro *run-off transcription reaction. Active complexes were pulled-down, washed, ran on a 4–20% SDS/PAGE, stained for the presence of histones and then western blotted for the presence of cyclin T_1 _or BRG1. Results in Figure 4D indicate that the wild-type Tat acetylated by HAT was able to efficiently bind to BRG1, consistent with results from panel B, where there was no HIV-1 DNA present in the reaction. Finally, to address whether these interactions were functionally significant in a chromatin transcription setting, we utilized the G_1_/S extract from CEM cells in an *in vitro *transcription reaction. Similar to HeLa cells (data not shown), CEM cells were treated with hydroxyurea/nocodazole, and samples were processed at 9 h post-release (G_1_/S) for *in vitro *transcription. The extracts were supplemented with SWI/SNF (all lanes), plus wild-type Tat (lanes 2 and 6), acetylated Tat (lanes 3 and 7), and mutant Tat (50/51) (lanes 4 and 8). Naked AdLuc DNA served as an internal control for transcription. As can be seen in Figure 4E, only acetylated Tat allowed for a robust transcription from the wild-type LTR (lane 3) and not the mutant TAR template (lane 7). Furthermore, addition of anti-acetyl Tat 50/51 antibodies (see Figure 5) at increasing concentrations (panel E, 100, 300, and 500 ng; lanes 10–12) specifically inhibited transcription from the HIV-1 template. Collectively, these results indicate that acetylayted Tat has a high affinity to BRG1, and a combination of both Tat and BRG1 (SWI/SNF) promote TAR-specific HIV-1 transcription.

### Presence of acetylated Tat and BRG1 on HIV-1 nucleosomal DNA *in vivo*

To assess the functional relevance of Tat acetylation *in vivo*, we performed a series of ChIP assays from HIV-1 infected cells. We first raised polyclonal antibodies against acetylated Tat using acetylated Tat peptides as antigens. These antibodies recognize Tat acetylation at lysine 41 or 50 and 51. Next, we transfected HLM-1 cells with a wild-type Tat plasmid (eTat). These cells are HIV-1^+^/Tat^-^, thus introduction of wild-type Tat allows for full *trans*-activation and viral progeny formation. When anti-acetyl-Tat antibodies were used for immunoprecipitation, we consistently observed an association of acetylated Tat at regions where nuc-1 is located but not nuc-0 (Figure 5). These data suggest that Tat is acetylated *in vivo *and is associated with the nuc-1 region of the HIV-1 promoter, which encompasses the transcription start site.

### Effect of BRG1 on HIV-1 transcription *in vivo*

**Figure 6:**
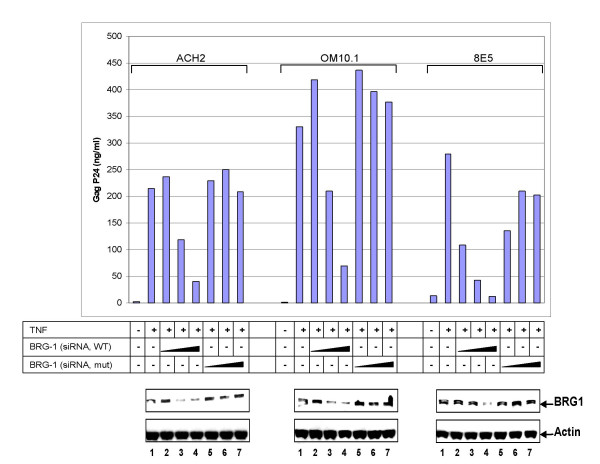
**Suppression of BRG1 expression by RNAi inhibits HIV-1 replication**. Complete sequence of BRG1 wild-type and mutant siRNA used in transfection. Oligos were designed and synthesized using the OligoEngine website [109]. HIV-1 infected cells (ACH_2_, OM10.1 and 8E5) were treated with TNF-α (10 ng/ml) for 2 h, washed, and subsequently electroporated (ACH_2_, [113]) or Amexa (OM10.1 and 8E5) treated with increasing amounts (1, 5, or 10 μg) of either wild-type (WT) or mutant (mut) BRG1 siRNA. Forty eight hours later, samples were collected and used for p24 gag ELISA. Western blot against BRG1 showed more than 90% decrease in protein levels in WT and not mutant BRG1 siRNA transfect cells [74].

Tat recruitment of SWI/SNF to the HIV-1 LTR presumably plays a role in the ability of Tat to *trans*-activate the promoter and induce replication. To assess this possibility, RNA interference (RNAi) of BRG1 expression in HIV-1 infected cells was used. Here, we synthesized a series of wild-type and mutant siRNA against BRG1. We chose five oligonucleotides that span the 5' end, middle, and 3' end of the BRG1 mRNA. The sequences of the siRNA and the nucleotide position are listed in Materials and Methods and have previously been published [74]. The most optimal sequences had a GC content of between 30%-70% [74]. To assess the importance of BRG1 in viral transcription, we utilized the HIV-1 chronically infected cell ACH_2_. Viral induction in ACH_2 _cells was achieved by treatment with TNF-α (Figure 6, left panel). Addition of increasing amounts of wild-type BRG1 siRNA (a mixture of all five oligonucleotides electroporated into cells) resulted in a decrease in p24 expression. However, when mutant BRG1 siRNA was utilized, p24 expression and, hence HIV-1 progeny formation, was unaffected. Similar results were obtained in other chronically infected cells including OM10.1, 8E5 (Figure 6, right panels), and J1-1 as well as U_1 _(data not shown). BRG1 protein expression was also decreased as a result of the siRNA (wild-type) treatment; mutant siRNAs did not affect BRG1 protein levels (Figure 6, bottom panel). These results indicate that BRG1 expression is critical for HIV-1 replication.

**Figure 7:**
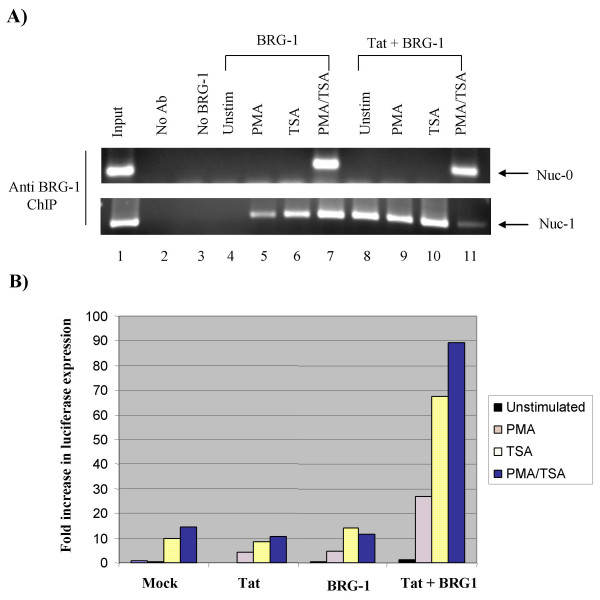
**BRG1 and Tat associate with chromatinized HIV-1 promoter in C33A cells at nuc-1**. **A) **The BRG1/hBrm-deficient cervical carcinoma cell line C33A was used for these experiments and maintained under standard conditions in Dulbecco's modified Eagle's medium. A BRG1 (10 μg) or Tat (3 μg) expression vector or a control vector (10 μg, lane 3) was transfected into C33A cells that have an HIV-1 reporter construct stably integrated into their genome. After cells were allowed to express BRG1 for 36 h, cultures were stimulated with either PMA for 2 h (lane 5), TSA for 12 h (lane 6), or both for 2 h and 12 h, respectively (lane 7), before ChIP. A ChIP assay was performed with BRG1 antibodies, and PCR was used to detect the recovery of nuc-0 and nuc-1 DNA. The input DNA represents the total genomic DNA. Ab, antibody; Unstim, unstimulated. **B) **BRG1 and Tat were transiently expressed in C33A cells that had been stably transfected with an HIV-1-luciferase construct. Thirty-six hours after transfection, cells were stimulated with either PMA for 2 h, TSA for 12 h, or both for 12 h. Following stimulation, cells were lysed, and the luciferase activity was measured [114]. Data presented is from average of two individual experiments.

Next, we investigated whether BRG1 was in fact recruited to the HIV-1 promoter *in vivo*. We transfected a BRG1 eukaryotic expression construct [74] into C33A cells that had an HIV-1 reporter construct stably integrated into its genome [75]. Cells were either left untreated, stimulated with PMA, treated with the HDAC inhibitor TSA, or exposed to both PMA and TSA. Following harvesting, a ChIP assay was performed with antibodies against BRG1. Immunoprecipitated DNA was analyzed by PCR with primers specific for nuc-0 and nuc-1. Figure 7A shows that BRG1 does interact with the nuc-0 region *in vivo *and that this interaction is dependent upon presence of both PMA and TSA (panel A top, lanes 7 and 11). The presence of BRG1 on nuc-0 was not Tat dependent (lanes 8–10). However, BRG1 was present on nuc-1 in the presence of PMA or TSA (panel A bottom, lanes 5, 6, 9, and 10). More importantly, BRG1 was present on nuc-1 in unstimulated cells in the presence of Tat (compare lanes 4 and 8). A Tat 50/51 mutant did not recruit BRG1 to the nuc-1 site (data not shown). Finally, since BRG1 is recruited to the nuc-1 region of the HIV-1 promoter in response to Tat expression alone, we investigated whether this recruitment stimulated transcription. C33A cells containing a stably integrated HIV-1 luciferase reporter construct were transiently transfected with the BRG1, Tat, or BRG1 and Tat expression constructs or a control vector (Mock). Figure 7B shows that without BRG1 or Tat; PMA, TSA or PMA and TSA stimulated HIV-1 luciferase transcription anywhere from 3- to 14-fold. However, in the presence of both Tat and BRG1 the stimulation increased from 28- to 89-fold above background, implying that to recover robust activated transcription, the integrated HIV-1 promoter requires both Tat and BRG1. This also indicates that a factor required for a high level of HIV-1 reporter expression is limiting in C33A cells. Hence, we concluded that Tat acetylation, followed by BRG1 recruitment, plays an important role in stabilizing the interaction between SWI/SNF and HIV-1 chromatin resulting in remodeling of nuc-1 and activated viral transcription.

### Effect of BRG1 and acetylated Tat on nucleosomes on the HIV LTR

A number of investigators, including the Verdin lab, have performed pioneering experiments to define the nucleosome positions in HIV-1 infected cells. The HIV-1 genome was first analyzed for the presence of DNase-I hypersensitive sites using an indirect end-labeling technique [45]. Three well characterized, chronically infected cell lines, ACH_2_, 8E5, and U_1_, were used to define nucleosome boundaries. Five major hypersensitive sites were identified, some of which were common among the three cell lines. They included DNase-I hypersensitive sites at the 5' LTR, namely HS2 (nt 223–325), HS3 (nt 390–449), and HS4 (nt 656–720) [46]. Both HS3 and HS4 are at the 5' and 3' boundaries of nucleosome-1 (nuc-1), respectively. Nuc-1 is located at the transcriptional start site and poses a block to activated transcription [76]. However, when activated transcription occurs, there is a disappearance of the periodic DNA protection pattern following digestion with DNase-I and reveals a profile of digested template essentially identical to naked DNA [46]. Therefore, nuc-1 is disrupted when activated transcription takes place in all three cell lines tested [45,46,76-80].

**Figure 8:**
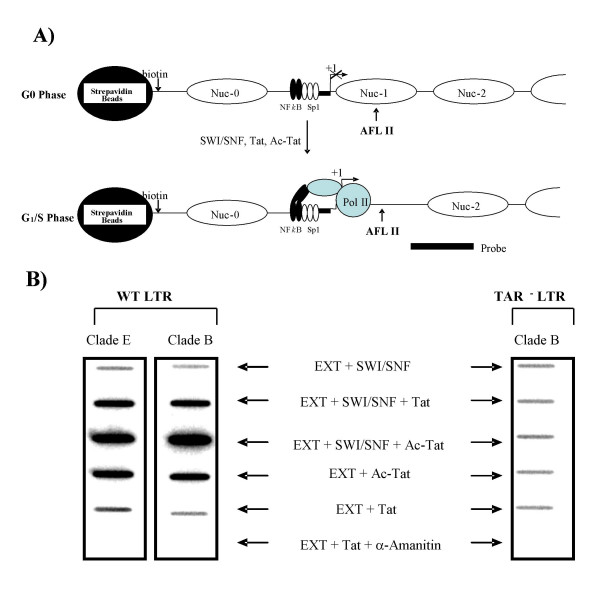
**Effect of SWI/SNF, Tat, or acetylated Tat on restriction enzyme accessibility**. **A) **A diagram of the transcription experiment. Immobilized templates (800 bp from the 5' U3 region and into the Gag region) were assembled into nucleosomes and SWI/SNF, Tat, or acetylated Tat were added to the reaction. In the absence of transcription, nuc-1 blocks restriction enzyme accessibility of *Afl II*. During transcription and remodeling of the nucleosome, the *Afl II *site becomes accessible, and the 3' end sequence is released. This fragment can be detected by southern slot blotting [74] using a probe (end labeled oligonucleotide) spanning the U5 region into Gag. **B) ***In vitro *transcription using CEM cells (G_1_/S extract). Similar to Figure 1, CEM cells were treated with hydroxyurea/nocodazole and samples were processed at 9 h post-release. The extracts (EXT, 200 μg) were supplemented with 200 ng of purified SWI/SNF, plus wild-type Tat (500 ng), or acetylated Tat (500 ng). α-amanitin at 0.1 μg/ml was used to detected RNAPII sensitivity. Clades B and E (a generous gift of J. Hiscott) and the TAR mutant LTR (TM26) were used as the DNA templates.

We then determined whether BRG1 and/or acetylated Tat are responsible for removing nuc-1, and whether we could faithfully mimic the *in vivo *data in an *in vitro *remodeling assay. We used two LTR sequences spanning 800 bp from the 5' U3 region into the Gag region. Sequences from clades B and E were PCR amplified with biotin-labeled primers, assembled into nucleosomes, and mixed with CEM G_1_/S extracts for *in vitro *transcription. To facilitate chromatin remodeling, we also added SWI/SNF, Tat, or acetylated Tat to the reaction. Following transcription, immobilized templates were pulled-down, washed, and used for restriction enzyme digestion (*Afl II*). *Afl II *cleaves at position 520 at the end of the R (TAR) region. The *Afl II *site is normally blocked if nuc-1 is present; however, the site becomes accessible if the nucleosome is remodeled or removed. Therefore, if *Afl II *is able to cleave the DNA, then a 3' fragment consisting of U5 and Gag will be released into the supernatant and the fragment can be detected by Southern analysis using a probe spanning the U5/Gag region. A diagram of the experiment is illustrated in Figure 8A while the results are shown in Figure 8B. The presence of CEM G_1_/S extracts and SWI/SNF allowed a low level of digestion with *Afl II*, and Tat increased accessibility by 3-fold (panel B, second line). However, in the presence of SWI/SNF and acetylated Tat, *Afl II *digestion was increased 13- to 15-fold for the LTRs of clades B and E (panel B, third line). The increase in digestion was specific to a combination of SWI/SNF and acetylated Tat, since neither complex alone dramatically increased accessibility. Finally, in the presence of a RNAPII inhibitor, α-amanitin, there was no *Afl II *digestion, indicating that active RNAPII transcription was needed for nuc-1 removal and *Afl II *accessibility. No increase in *Afl II *digestion was observed on the TAR mutant template (panel B, right side). Collectively, these results indicate that *in vitro *reconstitution of at least few nucleosomes on the LTR mimics the *in vivo *positioning of HIV-1 nucleosomes as seen in latently infected cells, and that the presence of SWI/SNF and acetylated Tat are sufficient to remodel/remove the blocking nuc-1 nucleosome on the HIV-1 LTR.

## Discussion

The chromatin structure presents a significant barrier to transcription. Various complexes, including HATs, covalently modify nucleosomal histone proteins through acetylation while ATP-dependent chromatin remodelers alter the chromatin structure via ATP hydrolysis. These modifications and alterations of chromatin structure increase DNA accessibility to transcription factors and activators thus promoting transcription initiation and efficient elongation. A more current view is that activators must first recruit chromatin remodelers in order to create a chromatin environment permissive for pre-inititation complex (PIC) assembly. Recently, it has become evident that other factors, such as the chromatin structure of the gene promoter and the phase of the cell cycle, also govern how chromatin remodelers collaborate with each other to control steps before, during, or after PIC assembly.

Although the precise nucleosome position of the integrated HIV-1 LTR is well characterized, there is little data about how this primary structure is folded into the chromatin fiber or other secondary structures (>30 nm fiber) and how these structures influence HIV-1 latency and transcriptional activation. Few studies have shown that the integration site and its corresponding chromatin environment affect HIV-1 gene expression [81,82]. Sequence analysis and mapping of HIV-1 integration sites in the cellular genome has provided evidence that chromatin structure plays an important role in subsequent HIV-1 transcription. Analysis of the site of integration during productive HIV-1 infection has shown that integration preferentially occurs in areas rich in retrotransposable Alu elements [83-85] and transcriptionally active genes (69% of 524 integration sites) [83]. These areas are characterized by increased chromatin accessibility and the accumulation of factors important for chromatin remodeling. These data are also consistent with earlier observations that sites which are preferentially accessible to DNase-I are favored for integration [86-89]. However, a small fraction of cells (less than 1%) exhibited post-integration latency, a state of transcriptional silencing.

Many reports in the last several years have linked Tat *trans*-activation to chromatin remodeling *in vitro *and *in vivo*. When transcription is activated by Tat in a LTR integrated cell line, the chromatin associated with sequences immediately downstream of the transcription start site become accessible to nucleases and the nucleosome adjacent to the transcription start site (nuc-1) becomes disrupted [78]. Similarly, transfection of Tat into Jurkat (Tat^-^) clones containing a integrated single HIV-1 mini-genome activated HIV-1 transcription and resulted in the disruption of nuc-1 to the same extent as TSA treatment [82]. Furthermore, immunoprecipitation of Tat from human cells identified a protein complex with HAT activity, suggesting that HATs are targeted to the HIV-1 promoter by Tat [90]. Interestingly, *in vitro *transcription from chromatinized templates indicated that Tat *trans*-activation is synergistic with Sp1 and NF-κB [48]. *In vivo*, HIV-1 LTR lacking Sp1 and NF-κB sites do not undergo chromatin remodeling and are unresponsive to Tat *trans*-activation [78]. Several other groups have observed that Tat is able to form a ternary complex with several HAT complexes, including p300/CBP, p/CAF, TAFII250, and Tip60, targeting these HAT proteins to the viral promoter [70,90-94].

Similar to our findings, two recent reports have shown the involvement of SWI/SNF as a cofactor for Tat activation of the HIV promoter. Tréand et al. [95] showed that, via its arginine-rich motif, Tat was able to interact with Brm, the enzymatic subunit of the SWI/SNF chromatin-remodeling complex. This interaction was regulated by Tat acetylation at lysine 50. Tat recruited the SWI/SNF complex to the LTR *in vivo*, leading to the activation of the integrated HIV-1 promoter. Another recent report from the Verdin lab also showed similar results [96]. Knockdown of INI-1 and BRG1, two components of the SWI/SNF chromatin-remodeling complex, suppressed Tat-mediated *trans*-activation, and cells deficient in INI-1 or BRG1 exhibited defective Tat *trans*-activation. Tat was found to be in complex with several SWI/SNF subunits, and the complex synergized with p300 to activate the HIV-1 promoter. A recent study from the Trono lab revealed that INI1 uses its repeat domains (Rpt 1 and 2) to bind and subsequently enhance the *trans*-activation potential of Tat [97]. While INI1 is dispensable for viral transduction, their findings suggest that incoming PIC might recruit INI1 to promote chromatin remodeling at the HIV-1 promoter and enhance transcription.

It is interesting to note that INI-1 (Snf5/BAF47) is also a potent tumor suppressor whose mechanism of action is largely unknown. Tumor suppressor activity of Snf5 depends on its regulation of cell cycle progression; Snf5 inactivation leads to aberrant up-regulation of E2F targets and increased levels of p53 that are accompanied by apoptosis, polyploidy, and growth arrest. Furthermore, conditional mouse models demonstrate that inactivation of p16Ink4a or Rb does not accelerate tumor formation in Snf5 conditional mice, whereas mutation of p53 leads to a dramatic acceleration of tumor formation [98]. Therefore, it would be interesting to further determine if the binding of Tat to the SWI/SNF complex (either through INI-1 or BRG1) could somehow control the expression of cell cycle genes (i.e., G_1_/S genes) and alter their activity. Current experiments are in progress to address this critical issue and whether G_1_/S genes such as various cyclins, including the cyclin E and T promoters, are modulated by the Tat/SWI/SNF complex.

We and others have shown that Tat can be acetylated at lysines 28, 50, and 51 leading to altered interactions between Tat and TAR, p300/CBP, and p/CAF, as well as activation of viral transcription and replication. They collectively support the notion that Tat first recruits p300/CBP to the HIV-1 promoter during initiation. Tat acetylated at lysine 50 then recruits p/CAF, which subsequently acetylates Tat at lysine 28. Acetylation of Tat at lysines 50 and/or 51 promotes the dissociation of Tat from TAR RNA and contributes to stimulation of transcription elongation [60,99]. The acetylated form of Tat is released from TAR, which can now bind to BRG1, a component of the SWI/SNF chromatin remodeling complex. We hypothesize that the interaction of Tat with BRG1 facilitates SWI/SNF to remodel downstream nucleosomes allowing for further transcription of the HIV-1 genome. We propose that the major function of the interactions between Tat and bromodomain proteins is to modify HIV-1 chromatin such that the LTR becomes responsive to Tat and allows ample and rapid activated transcription to occur. This also may set the stage for re-initiation of transcription where pre-made (activated) complexes may be recycled for HIV-1 activated transcription. Future experiments will address these particular issues along with Tat's effect on the remodeling activity of the SWI/SNF complex at the proximal promoter and ORF regions of HIV-1 genome.

## Materials and methods

### Cell culture

ACH_2 _and 8E5 cells are both HIV-1-infected lymphocytic cells, with a single integrated wild-type copy (ACH_2_) and a single integrated copy containing a defective reverse transcriptase (8E5) in CEM (12D7) cells. The CEM T cell (12D7) is the parental cell for both ACH_2 _and 8E5 cells. U_1 _is a monocytic clone harboring two copies of the viral genome from parental U973 cells. All cells were cultured at 37°C with up to 1 × 10^5 ^cells per ml in RPMI 1640 media containing 10% fetal bovine serum (FBS), 1% streptomycin/penicillin antibiotics, and 1% L-glutamine (Gibco-BRL, Carlsbad, CA). HLM-1 cells (AIDS Research and Reference Reagent Program, Catalog No. 2029) were derived from HeLa-T41 cells integrated with one copy of the HIV-1 genome containing a Tat-defective mutation. The mutation was introduced as a triple termination linker at the first AUG of the Tat gene. HLM-1 cells are completely negative for virus particle production, but can be induced to express one cycle of infectious HIV-1 particles after transfection with Tat cDNA or mitogens such as TNF-α or sodium butyrate. HLM-1 cells were grown in DMEM containing 10% FBS, 100 mg/ml of G418, plus 1% streptomycin/penicillin, and 1% L-glutamine (Gibco BRL). Cells were grown to 75% confluency prior to the transfection. Plasmids were transfected into HLM-1 cells by electroporation or Amexa, washed after 4 h, and re-fed with fresh complete DMEM with 10% FBS for the remainder of the experiment.

### Generation of epitope-tagged Tat-expressing cell line

HeLa CD4^+ ^cells were used for transfection with either an epitope-tagged (the influenza epitope at the C-terminus of Tat 1–86) plasmid or the parental vector pCEP4. Following transfection, cells were selected with 200 μg of hygromycin/μl. Hygromycin-resistant lines established from single-cell clones were maintained for up to 12 months with continuous passage and used to make extracts for *in vitro *transcription analysis [53].

### ChIP assays

Five to ten million infected cells in log phase were incubated for 2 h with or without 5 μg/ml TNF-α to induce transcription of latent proviral DNA. Cells were subsequently left untreated or treated with siRNA. After 48 h, cells were cross-linked (1% formaldehyde, 10 min at 37°C) and samples were sonicated to reduce DNA fragments to 200–800 nt lengths for ChIP assays. Specific transcription complexes were immunoprecipitated with appropriate antibodies. DNA sequences in the immunoprecipitates were detected by PCR using primers specific for the HIV-1 LTR (forward primer, 5'-ACTTTTCCGGGGAGGCGCGATC-3'; reverse primer, 5'-GCCACTGCTAGAGATTTCCACACTG-3') or Env region (forward primer, 5'-CCTTG(T)GAGCCAATTCCCATA-3'; reverse primer, 5'-TAACAAATGCTCTCCCTGGTC-3').

### MALDI-TOF analysis

Individual protein bands were excised from the silver-stained gel and destained with a solution of 30 mM potassium ferricyanide/100 mM sodium thiosulfate (1:1) (v/v). Trypsin-digested sample solutions were further desalted and concentrated with C_18 _ZipTips (Millipore, Framingham, MA). Samples were mixed with the same volume of the matrix solution (α-cyano-4-hydroxycynnamic acid in 50% acetonitrile/0.1% [v/v] trifluoroacetic acid). Two microliters of the mixtures were applied to the sample plate and introduced into the mass spectrometer after drying. Mass spectra were recorded in the reflectron mode of a MALDI-TOF mass spectrometer (Voyager-Elite; PerSeptive Biosystems) by summing 200–300 laser shots with an acceleration voltage of 20 kV, 70% grid voltage, 0.05 guide wire voltage, 100 ns delay, and low mass gate at 700 *m*/*z*. Proteins were identified using the peptide mass fingerprinting analysis software ProFound [101]. The NCBInr database was used for the searches with several passes of searching with different limitations for each spot. In general, all bands were searched using methionine oxidation and no limitation for pI as criteria. The most optimal match for each spot was considered using higher coverage rate, more matched peptides, and higher score without limitations on the taxonomic category and protein mass. Zero missed cleavage by trypsin and lowest mass tolerance, *i.e*. ± 50 ppm, were considered for most of the proteins. A few bands were searched with the following parameters to find the best match: two missed cut cleavages, limited to the "mammal" category, and/or a set ± 50% of total molecular mass. We consistently used multiple parameters such as low miss cut, low ppm, and first methionine oxidation in our searches to obtain reliably matched proteins. Identified proteins (i.e., Sp1, TFIIB, cdk9, Tat, and BRG1) were confirmed by Western blot analysis. Unless stated specifically, all data bases and tools used for bioinformatics analysis were from the following public websites: PubMed [102], ExPASy [103], BLAST [104], Pfam [105], PBIL [106], COILS [107], and PIR [108].

### siRNA analysis

Oligonucleotides were designed and synthesized using the OligoEngine website [109] and the accession number for BRG1. Five oligonucleotides (see below), which span the 5' end, middle, and 3' end of the BRG1 mRNA, were chosen. The most optimal sequences had a GC content between 30% and 70%. HIV-1 infected cell lines were treated with TNF-α for 2 h. A mixture of the five oligonucleotides was electroporated into the cells, and HIV-1 replication was monitored by p24 Gag enzyme-linked immunosorbent assay (ELISA). The sequences of oligonucleotides used for BRG1 siRNA (wild-type) were as follows: [GenBank:U29175] -1716, GGACAAGCGCCUGGCCUAC; [GenBank:U29175] -2142, GAAGAUUCCAGAUCCAGAC; [GenBank :U29175] -3210, GAUCUGCAACCACCCCUAC; [GenBank:U29175] -4236, GCAGUGGCUCAAGGCCAUC; [GenBank:U29175] -4776, GGAGGAUGACAGUGAAGGC. The sequences used for BRG1 siRNA (mutant) were as follows: [GenBank:U29175] -1716, GGACAAAAAAAUGGCCUAC; [GenBank:U29175] -2142, GAAGAUUCCAAAAAAAGAC; [GenBank:U29175] -3210, GAUCUGCAACCAAAAAUAC; [GenBank:U29175] -4236, GCAGUGGCUCAAAAAAAUC; [GenBank:U29175] -4776, GGAGGAUGAAAAAAAAGGC.

### Cell cycle analysis

The eTat, control (pCEP4), CEM, and OM10.1 cells were either blocked with hydroxyurea for 18 h or blocked with hydroxyurea (2 mM final concentration), washed, and released for 1 h, followed by addition of nocodazole (50 ng/ml) for 14 h. Following the block, the cells were washed twice with phosphate-buffered saline (PBS) and released with complete medium. Samples were collected every 3 h, and the cells were used to make whole-cell extracts (5 × 10^7 ^cells/time point) for *in vitro *transcription or Western blot analysis or processed for fluorescence-activated cell sorting (FACS). Single-color flow cytometric analysis of DNA content was performed on various cell lines. The cells were washed with PBS, and approximately 2 × 10^6 ^cells were fixed with 500 μl of 70% ethanol. The cell pellets were washed three times with PBS and incubated in 1 ml of PBS containing 150 μg of RNase A (Sigma)/ml and 20 μg/ml of propidium iodide (Sigma, St. Louis, MO) at 37°C for 30 min. The stained cells were analyzed for red fluorescence (FL2) on a FACScan (Becton Dickinson, San Jose, CA), and the distribution of cells in the G_1_, S, and G_2_/M phases of the cell cycle was calculated from the resulting DNA histogram with Cell FIT software, based on a rectangular S-phase model.

### *In vitro *transcription

*In vitro *transcription was performed with HeLa or CEM whole-cell extracts (25 to 50 μg total) on immobilized HIV-1 LTR chromatin templates which were assembled as described below. The DNA fragments were biotinylated, gel purified, and reconstituted with core histones by step dilution. Briefly, core histones were purified from HeLa cells and mixed with DNA. The biotinylated mononucleosome were prepared by mixing the biotinylated DNA and purified core histones by sequential dilution from 1 to 0.1 M NaCl and subsequently phased. The biotinylated nucleosomal arrays were then incubated at 30°C for 1 h with paramagnetic beads coupled to streptavidin in a binding buffer containing 10 mM HEPES (pH 7.8), 50 mM KCl, 5 mM DTT, 5 mM phenylmethylsulfonyl fluoride, 5% glycerol, 0.25 mg/ml bovine serum albumin (BSA), and 2 mM MgCl_2_, supplemented with 300 mM KCl. *In vitro *transcription reactions were incubated for 1 h at 30°C and contained the nucleoside triphosphates ATP, GTP, and CTP at a final concentration of 50 μM and [^32^P]UTP (20 μCi; 400 Ci/mmol; Amersham, Piscataway, NJ) in buffer D (10 mM HEPES [pH 7.9], 50 mM KCl, 0.5 mM EDTA, 1.5 mM dithiothreitol, 6.25 mM MgCl_2_, and 8.5% glycerol). Transcription reactions were terminated by the addition of 20 mM Tris-HCl (pH 7.8), 150 mM NaCl, and 0.2% SDS. The quenched reactions were extracted with equal volumes of phenol-chloroform and precipitated with 2.5 volumes of ethanol and ^1^/_10_volume of 3 M sodium acetate. Following centrifugation, the RNA pellets were resuspended in 8 μl of formamide denaturation mix containing xylene cyanol and bromophenol blue, heated at 90°C for 3 min, and seperated at 400 V in a 10% polyacrylamide (19:1 acrylamide-bisacrylamide) gel containing 7 M urea (pre-ran at 200 V for 30 min) in 1 × Tris-borate-EDTA. Transcript sizes of 250 nt for AdLuc and 375 nt for HIV-1 transcripts were observed. The gels were analyzed with the Molecular Dynamics PhosphorImager screen and radioactivity was quantitated with ImageQuant.

### Immunofluorescence staining by flow cytometry

OM10.1 cells were used to analyze HIV-1 gene expression at various stages of the cell cycle. Following arrest of cells at G_0 _by serum starvation, cells were washed in Hanks' balanced salt solution and fixed in 70% ice-cold ethanol. After fixation, cells were rehydrated in PBS containing 1% BSA and aliquots containing 0.5 × 10^6 ^to 1 × 10^6 ^cells were resuspended in 150 μl of PBS-BSA. The abundance of Nef, Env, cyclin E and cyclin A were determined after immunofluorescence staining [56]. Cells were stained with mouse anti-human monoclonal antibodies to cyclin E (1:100; SC, clone HE67), cyclin A (1:50; SC, clone BF683), Nef (1:50; AIDS reagent catalog, EH1), and Env (1:50; AIDS reagent catalog, 48 d) or a nonspecific immunoglobulin (DAKO) overnight. Samples were washed in PBS-BSA and stained with a fluorescein isothiocyanate-labeled goat anti-mouse secondary antibody (1:30; DAKO) for 30 min in the dark.

## Abbreviations

Abbreviations: BAF, BRG1 associated factors; BRG1, Brahma related gene-1; HAT, histone acetyltransferase; HDAC; histone deacetylase; HIV-1, human immunodeficiency virus type 1; LTR, long terminal repeat; Nuc, nucleosome; SWI/SNF, Switching/Sucrose Non-Fermenting; Tat, Transactivator.

## Competing interests

The author(s) declare that they have no competing interests.

## Authors' contributions

EA performed the *in vitro *transcription and p24 assays. LD performed experiments in Figures 2, 3, 4, 5, 6, 7. LOD performed the BRG1 western blot on siRNA treated cells and revised the manuscript. AP assisted in designing the experiments. FK guided the experiments and drafting of the manuscript.
